# LINC00525 enhances ZNF460-regulated CD24 expression through the sponge miR-125a-5p to promote malignant progression of breast cancer

**DOI:** 10.1007/s00432-024-05830-2

**Published:** 2024-06-24

**Authors:** Jun Wang, Ji Shi, Yuan Xiang, Zhi-Wen Wang, Fei-Fei Qi, Zi-Yi Li, Li-Li Zhao, Guan-Hua Zhu, Yuan-Yuan Duan, Zhong-Yi Yang, Jia-Peng Li, Xing-Hua Liao

**Affiliations:** 1 Institute of Biology and Medicine, College of Life and Health Sciences, Wuhan University of Science and Technology, Wuhan, People’s Republic of China; 2 Department of Medical Laboratory, Central Hospital of Wuhan, Tongji Medical College, Huazhong University of Science and Technology, Wuhan, China; 3 Key Laboratory of Chronic Noncommunicable Diseases, Yueyang Vocational Technical College, Yueyang, China; 4 Yueyang Engineering Technology Research Center of Breast Disease Diagnosis and Treatment, Yueyang People’s Hospital, Yueyang Hospital, Affiliated to Hunan Normal University, Yueyang, China

**Keywords:** CD24, ZNF460, miR-125a-5p, LINC00525, Proliferation, Migration

## Abstract

**Introduction:**

CD24 is a highly glycosylated glycosylphosphatidylinositol anchored membrane protein that plays an important role in tumor progression. The aim of this study was to investigate the effect of abnormal expression of CD24 on the proliferation, migration and invasion of breast cancer (BC) cells, and the molecular mechanism of regulating CD24 expression in breast cancer.

**Methodology:**

The bioinformatics method was used to predict the expression level of CD24 in BC and its relationship with the occurrence and development of BC. IHC, RT-qPCR and WB were used to detect the expression of CD24 in BC tissues and cells. The proliferation of CD24 was evaluated by CCK-8 and colony formation assay, and the migration and invasion of CD24 were evaluated by wound healing and transwell. In addition, the effect of CD24 on the malignancy of BC in vivo was further evaluated by subcutaneous tumorigenesis assay. Molecular mechanisms were measured by luciferase reporter assays, biotin-labeled miRNA pull-down assay, RIP, and western blotting.

**Results:**

The results show that CD24 is highly expressed in breast cancer tissues and cell lines, and knockdown of CD24 in vivo and in vitro can inhibit the proliferation, migration and invasion of BC cells. Mechanistically, the transcription factor ZNF460 promotes its expression by binding to the CD24 promoter, and the expression of ZNF460 is regulated by miR-125a-5p, which inhibits its expression by targeting the 3’UTR of ZNF460. In addition, LINC00525 acts as a ceRNA sponge to adsorb miR-125a-5p and regulate its expression.

**Conclusions:**

Overexpression of CD24 is involved in the development and poor prognosis of BC, which can be used as a potential target for the treatment of BC and provide a theoretical basis for the treatment of BC.

**Supplementary Information:**

The online version contains supplementary material available at 10.1007/s00432-024-05830-2.

## Introduction

Breast cancer (BC) is the most common malignancy in women worldwide, accounting for 30% of all female cancer cases and 15% of all female cancer deaths, and is the leading cause of cancer death in women (Sung et al. 2021; Siegel et al. 2023). Although there have been some advances in the early diagnosis, surgical treatment, endocrine therapy, radiotherapy, chemotherapy and targeted therapy of BC in the past few decades, there are still many patients facing the threat of recurrence and death (Fernandes et al. 2023). Therefore, it is of great significance to explore the molecular mechanism of BC occurrence and development and to screen new diagnostic and therapeutic targets.

CD24 is a small glycosyl phosphoinositol protein composed of 31 amino acids, is a mucin-like cell surface protein depending on the cell or tissue type, which may account for the functional diversity of CD24 (Kristiansen et al. 2004; Altevogt et al. 2021; Huth et al. 2021). The absence or presence of CD24 may affect the composition of the membrane raft, which in turn affects transcription factors and signaling pathways, which play a key role in the body's ability to fight off cancer cells (Kristiansen et al. 2004). Studies have shown that CD24 is widely expressed in various malignant tumors such as gastric cancer, ovarian cancer, liver cancer and lung cancer (Wang et al. 2014; Tarhriz et al. 2019; Li et al. 2019; Qiao et al. 2021). CD24 is highly expressed among various tumor cells, which is closely related to the development of tumors. Studies have shown that the abnormal expression of CD24 has promoted the proliferation, migration and invasion of liver cancer cells (Li et al. 2019). ELF5, as a transcription factor, induces CD24 expressions at the transcription level, CD24 high expression promotes the proliferation and invasion of cancer cells (Qu et al. 2021). CD24 is considered a symbol that affects the progress of osteosarcoma and is related to the prognosis of osteosarcoma (Zhou et al. 2020). Therefore, the targeted therapy of CD24 may be a promising treatment strategy for treating BC.

Transcription factors (TFS) play an important role in the regulation of gene expression of tumorigenesis, migration and invasion by binding specific sequences to promote or inhibit the expression of downstream genes (Bushweller 2019). Zinc finger protein (ZFP) is a class of transcription factors with finger-like DNA-binding domains that can directly recognize or bind DNA sequences to regulate gene transcription and play a role in many biological processes (Bu et al. 2021). Previous studies have shown that ZNF460 contributes to the occurrence and development of tumors. For example, ZNF460 up-regulates COMMD7 to promote the proliferation of acute myeloid leukemia (Shao et al. 2023), and ZNF460 mediates epithelial-mesenchymal transformation through trans-activation of APOC1 expression to promote the progression of gastric cancer (An and Liu 2023). In addition, ZNF460 up-regulates the expression of LINC00857 and accelerates the progression of pancreatic adenocarcinoma (Zhang et al. 2022). However, there have been no reports on the correlation of ZNF460 in BC.

MicroRNA (microRNA/miRNA) is a small fragment of single-stranded non-coding RNA about 22 nucleotides long that binds to the complementary sequence of the 3 'untranslated region (3'UTR) of target messenger RNA (mRNA), causing mRNA degradation or inhibiting its translation, and regulating gene expression at the post-transcriptional level (Papadaki et al. 2018). In addition, miRNA can interact with long non-coding RNA (lncRNA). LncRNA act as competitive endogenous RNAs (ceRNAs) to inhibit miRNA expression by acting as “molecular sponges” of miRNA (Poliseno et al. 2010). More and more types of miRNAs have been shown to be involved in tumor initiation and development, including regulation of cell proliferation, genomic stability, apoptosis, invasion, and angiogenesis (Lin and Gregory 2015; Gajos-Michniewicz and Czyz 2019). MiR-125a-5p has been confirmed to be involved in the progression of various cancers. Studies have shown that miR-125a-5p can promote the growth and invasion of gastric cancer by regulating the hippo pathway (Li et al. 2021), and miR-125a-5p can inhibit the progression of bladder cancer by targeting FUT4 (Zhang et al. 2018). In addition, MiR-125a-5p functions as a tumour suppressor in breast cancer by downregulating BAP1 (Yan et al. 2018).

In this study, we report that CD24 is highly expressed in BC and is used as an independent predictor of overall survival. In addition, CD24 is a direct transcriptional target of ZNF460, and CD24 regulates the proliferation, migration, and invasion of BC cells in vitro and in vivo. Mechanistically, LINC00525 may regulate CD24 expression by acting as a competitive endogenous RNA (ceRNA), and inhibit the degradation of ZNF460 by competing with miR-125a-5p, thereby inhibiting the degradation of CD24. In conclusion, our findings suggest that CD24 plays an important role in the malignant progression of BC. It provides important experimental evidence for the diagnosis and treatment of BC.

## Materials and methods

### Patients and tissue samples

The fresh tumor tissues and para-cancer tissues of 16 BC patients were obtained from Tongji Hospital, Tongji Medical College, Huazhong University of Science and Technology. All patients did not receive chemotherapy or radiotherapy prior to surgery and signed written informed consent. The study was reviewed and approved by the Hospital Ethics Committee of Huazhong University of Science and Technology, and all subjects signed the informed consent form of Helkisian.

### Cell lines

BC cell lines MDA-MB-231 and MCF7 were purchased from the Wuhan typical culture collection center (CCTCC), and normal breast epithelial cells MCF10A were purchased from Wuhan Procell Life Science&Technology Co.,Ltd. (Wuhan, China). DMEM cell medium containing fetal bovine serum (FBS) and MCF10A special medium were used and cultured in cell incubators under conditions of 37℃, 5% CO_2_.

### RT-qPCR

The mRNA expression levels of CD24 and other genes in BC tissues and cells were detected using RT-qPCR according to the manufacturer's instructions. β-actin was used as a control. Total RNA was extracted using the ultrapure RNA extraction Kit (Yeasen, Shanghai, China), and cDNA was synthesized using the ABScript II cDNA First Strand Synthesis Kit for the QPCR Kit (Abclone, Wuhan, China).

For miRNA quantification, RiboBio Co., Ltd. (Guangzhou, China) designed a convex-ring TM miRNA RT-qPCR primer set specifically for miR-125a-5p. CDNA was synthesized using miRNA first strand cDNA synthesis kit (Vazyme, Nanjing, China).

RT-qPCR was performed with the cDNA obtained in the above steps as a template, and three replicate wells were set in each group. PCR was performed for 40 cycles of 5 min at 95 ℃, 12 s at 95 ℃, 20 s at 58 ℃ and 20 s at 72 ℃.

The primers were listed in supplementary Table 1.

### Western blot (WB)

Cells were lysed with protease inhibitor RIPA lysis buffer (Meilun, Wuhan, China) ice. Protein extracts (30 μg) were electrophoretically separated on SDS–polyacrylamide gel and then transferred to polyvinylidene fluoride (PVDF) membranes, blocked with 5% milk for 1 h at room temperature, and then incubated with antibodies overnight. Secondary antibodies (1:5000, AS014, ABclone, Wuhan, China) were added, incubated for 1 h at room temperature and imaged on a chemiluminescent imaging system. The following antibodies were used: anti-β-actin (1:5000, ABclone, AB clone, Wuhan, China), anti-CD24 (1:1000, AB290730, ABCAM, Cambridge, UK), anti-Ki67(1:1000, AC002, ABclone, Wuhan, China, anti-Vimentin (1:1500,10,366–1-AP, proteintech, Wuhan, China), anti-E-cadherin (1:1000, A20798, ABclone, Wuhan, China). Anti-ZNF460 (1:1000, A17621, ABclone, Wuhan, China).

### Cell Counting Kit-8 (CCK-8) assay

The proliferation ability of BC cells was evaluated by the CCK-8 method (Meilun, Wuhan, China). 5 × 10^3^ cell suspensions were inoculated into 96-well culture plates containing 100 μL medium. 10 μL CCK-8 reagent was added to each well after 1, 2, 3, 4 and 5 days, respectively. After incubation at 37℃ for 1 h, absorbance was determined at 450 nm.

### Colony formation assay

Colony formation assays were performed to test the clonal ability of BC cells. The 1 × 10^3^ cells were cultured in 6-well plates for 14 days, and the medium was changed every 5 days. The colonies were fixed with 4% paraformaldehyde and stained with 0.1% crystal violet for 15 min. Colony Formation is determined by counting the number of stained colonies.

### Cell migration and invasion

Wound healing assays were used to test cell migration. A total of 2 × 10^5^ cells were seeded in 6-well plates, scraped with sterile plastic tips after 12 h of culture, washed with medium, and then cultured for 24 h and 48 h. At different time points, images of the plates were obtained using an inverted microscope.

For the invasion assay, 1 × 10^5^ cells in serum-free medium were placed in the upper chamber coated with matrigel. The medium containing 10% FBS was added to the lower chamber. After incubation for 24 h, the residual cells on the upper membrane were removed with absorbent cotton. Cells that had migrated or invaded the membrane were fixed with paraformaldehyde and stained with 0.1% crystal violet. Take an image using an inverted microscope.

### Luciferase reporter assays

Binding motifs to ZNF460 in the CD24 promoter region were identified by JASPAR (http://JASPAR.genereg.net/). The complete and mutated CD24 promoter sequences were synthesized and then inserted into the pGL3-basic vector and cotransfected into 293 T cells with the ZNF460 plasmid. MiR-125a-5p mimics were cotransfected into 293 T cells with wild-type and mutant LINC00525 plasmids. All vectors were validated by sequencing and luciferase activity was assessed using a dual luciferase detection kit (Yeasen, Shanghai, China) according to the manufacturer's instructions.

### Biotin-labeled miRNA pull-down assay

Biotin-labeled miR-125a-5p and miRNA control were transfected into BC cells and lysis were harvested 48 h later. The Streptavidin Dynabeads (Merck, Darmstadt, Germany) were washed and resuspended in buffer. Equal volumes of biotin-labeled miR-125a-5p and miRNA control were then added to the buffer. After incubation at room temperature for 10 min, the coated beads were separated with a magnet and washed 3 times. The isolated RNA was then analyzed by RT-qPCR.

### RNA immunoprecipitation (RIP)

An EZMagna RNA immunoprecipitation (Millipore, Bedford, MA, USA) was used according to the manufacturer's protocol. MCF-7 and MDA-MB-231 cells were lysed in RIP lysis buffer (containing protease inhibitor and phosphatase inhibitors) for 30 min, the cell extracts were incubated with magnetic beads conjugated with specific antibody or control IgG at 4 ℃ overnight. The magnetic beads were washed and incubated with protease K to remove the protein. Finally, the purified RNA was analyzed by RT-qPCR.

### Establishment of xenografts and in vivo studies

4-week-old female BALB/C nude mice were bred under specific pathogen-free conditions and operated according to protocols approved by the Ethics Committee for Wuhan University of Science and Technology animal experiments. MCF7 cells stably transfected with 1 × 10^7^ control shRNA or sh-CD24 were injected subcutaneously into one side of the nude mice. Tumor volume was measured with a caliper and recorded every 5 days. Euthanasia was performed after 25 days of growth, and tumor tissue was excised for subsequent experiments.

### Statistical analysis

GraphPad Prism 8 software was used to plot and analyze the original data. The statistical results were the results of three independent repeated experiments unless otherwise stated *test* for differences in two different sets of data using unpaired Student's-T test, and analysis of differences between multiple sets of data using one-way ANOVA. In vitro experimental data were expressed as mean ± standard deviation. A *P* value of less than 0.05 was considered statistically significant.

## Result

### High expression of CD24 in BC is associated with poor prognosis

**Fig. 1:**
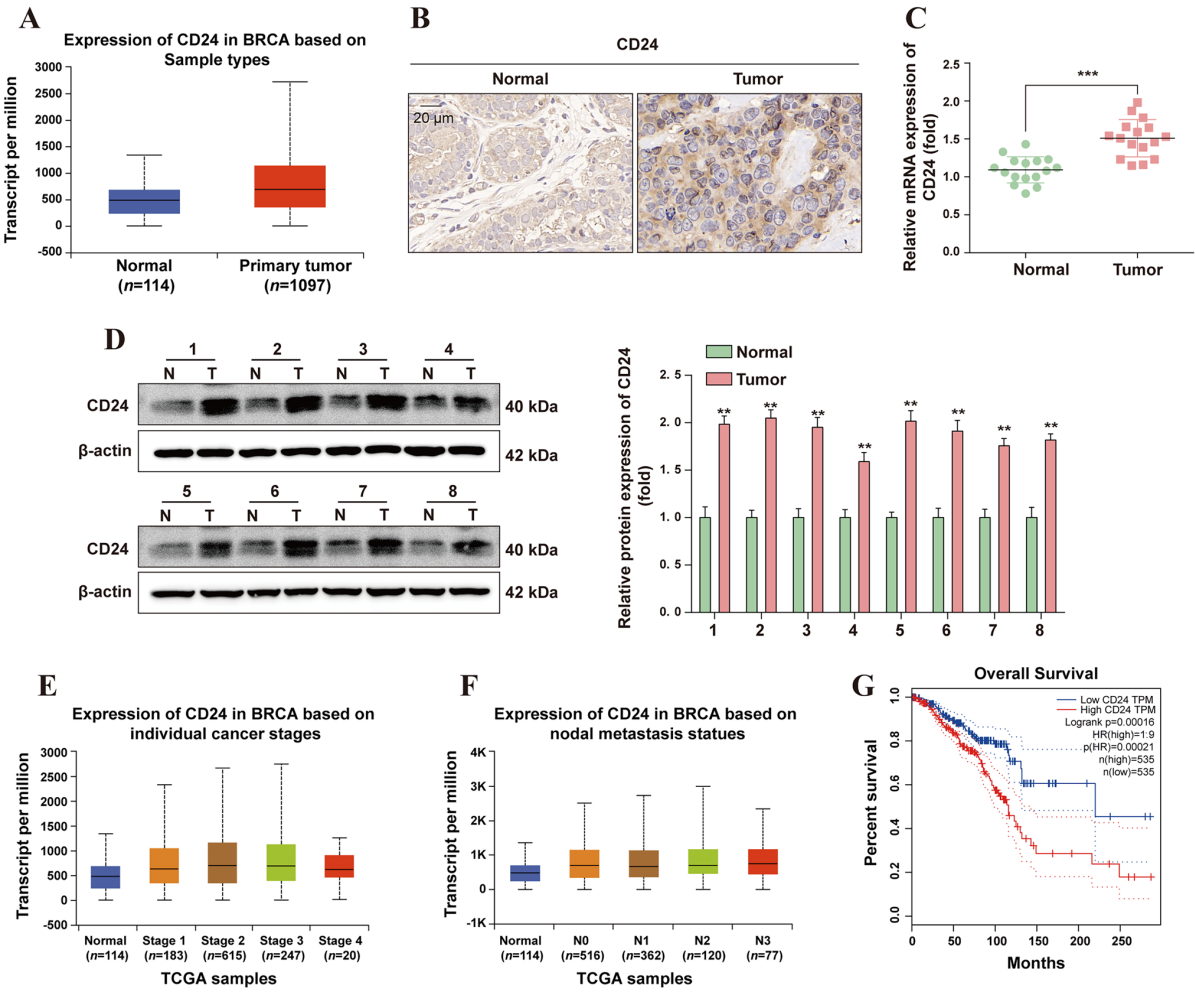
High expression of CD24 in BC is associated with poor prognosis. **A** UCLAN database (https://ualcan.path.uab.edu/index.html) was used to analyze the expression level of CD24 in BC. **B** The expression level of CD24 in BC tissues and adjacent tissues was detected by IHC. **C** The expression level of CD24 mRNA in BC tissues and adjacent tissues was determined by RT-qPCR (*n* = 16). **D** The expression level of CD24 protein in BC tissues and adjacent tissues was detected by WB, and the WB signals were normalized with β-actin (*n* = 8). **E** The UCLAN database (https://ualcan.path.uab.edu/index.html) was used to predict the relationship between CD24 expression levels and cancer stage. **F** The UCLAN database (https://ualcan.path.uab.edu/index.html) was used to predict the correlation between CD24 expression levels and nodal metastasis status. **G** The GEPIA database (http://gepia.cancer-pku.cn/) was used to predict the relationship between CD24 expression levels and survival time in BC patients. * *, *P* < 0.01; * * *, *P* < 0.001

To assess CD24 expression level in BC, UCLAN database (http://Starbase.sysu.edu.cn/index.php) was used to predict CD24 expression level, the result shows that compared with the normal tissue, mRNA expression levels of CD24 were elevated in BC tissues (Fig. 1A). To validate this analysis, we collected tumor tissues from 16 HCC patients for IHC and pathological analysis. The results of IHC showed that CD24 was highly expressed in BC tissues, while the expression level was low in paracancer tissues (Fig. 1B). According to the IHC staining score, patients were divided into low CD24 (*n* = 5) and high CD24 expression groups (*n* = 11). The results showed that CD24 expression was closely related to histological type (*P* = 0.045), primary tumor classification (*P* = 0.014) and TNM stage (*P* = 0.032) (Supplementary Table 2). To further demonstrate the high expression of CD24 in BC, RT-qPCR and WB detected the mRNA and protein levels of CD24, and the results showed that the mRNA and protein levels of CD24 were highly expressed in BC tissues compared with para-cancerous tissues (Fig. 1C, D). The results of bioinformatics analysis showed that high expression of CD24 was associated with poor prognosis in BC patients. CD24 expression level has a significant positive correlation with cancer staging and nodal metastasis status (Fig. 1E, F). Increased CD24 level predicts poor overall survival (OS) in BC patients (Fig. 1G). These results suggest that CD24 is up-regulated in BC and is associated with the malignant development of BC.

### The high expression of CD24 promotes the proliferation, migration and invasion of BC cells

**Fig. 2:**
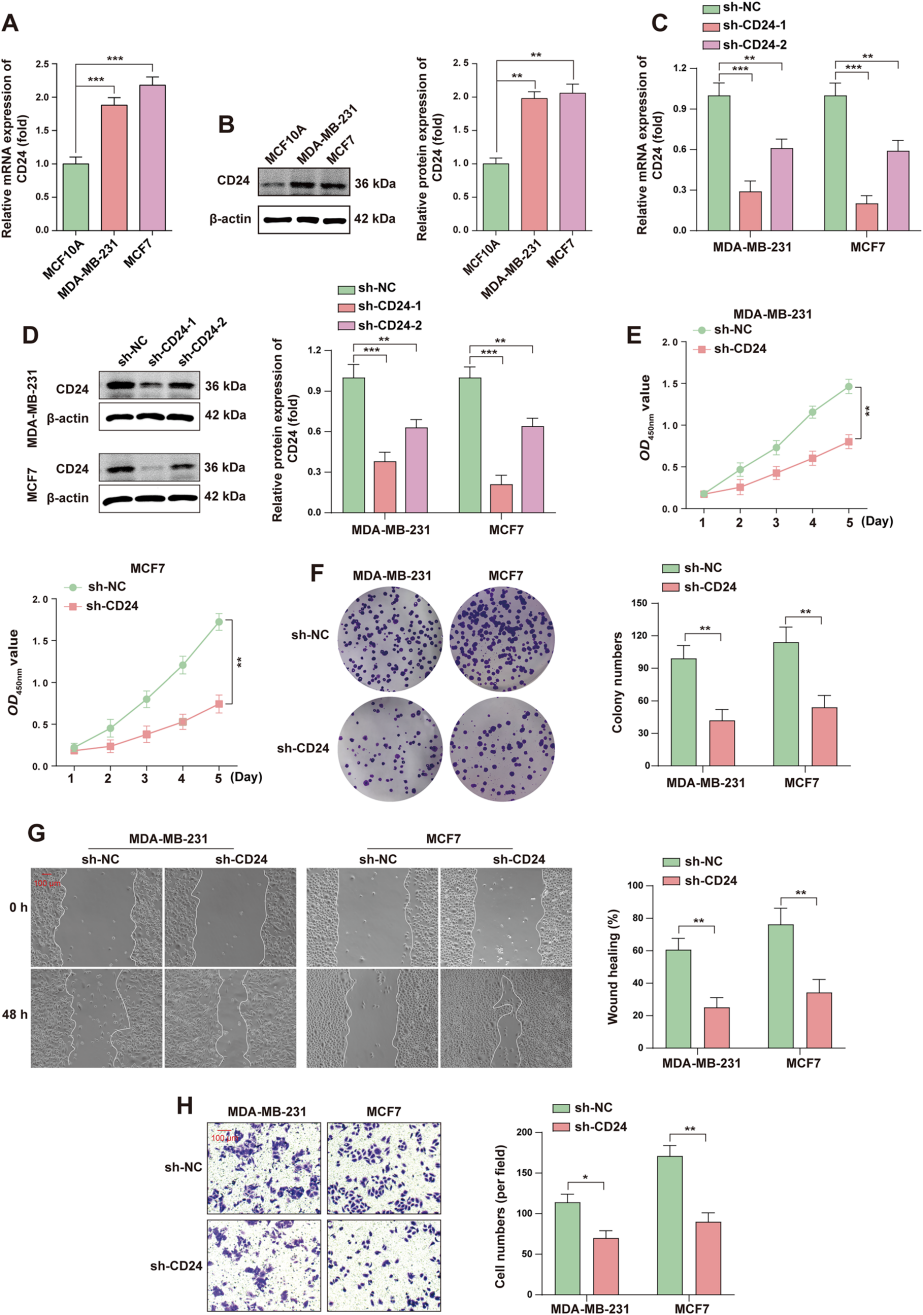
The high expression of CD24 promotes the proliferation, migration and invasion of BC cells. **A** RT-qPCR assay was used to detect CD24 mRNA levels in MDA-MB-231, MCF7 and MCF10A cells. **B** The protein levels of CD24 in BC cells and MCF10A cells were detected by WB assay and the WB signals were normalized with β-actin. **C** RT-qPCR assay was used to detect the knockdown efficiency of CD24 on mRNA. **D** WB assay was used to detect the knockdown efficiency of CD24 at the protein level in BC cells and the WB signals were normalized with β-actin. **E** CCK-8 assay was used to evaluate the effect of CD24 knockdown on BC cell viability. **F** The representative results of colony formation were determined by BC cells knocking down CD24, and the experimental results were analyzed statistically. **G** The migration ability of BC cells after CD24 knockdown was evaluated by wound healing experiment, and the results were statistically analyzed. **H** Transwell assay was used to evaluate the invasion ability of CD24 knockdown cells and the results were statistically analyzed. *, *P* < 0.05; * *, *P* < 0.01; * * *, *P* < 0.001

The expression of CD24 in BC cell lines (MDA-MB-231 and MCF-7) was significantly higher than that in normal breast epithelial cells MCF-10A (Fig. 2A, B). A BC cell line with stable knockdown of CD24 was successfully constructed to further explore the biological function of CD24 (Fig. 2C, D). Next, CCK-8 was used to detect cell viability, and the results showed that the viability of cells decreased significantly after CD24 knockdown (Fig. 2E). In addition, the results of colony formation assay showed that CD24 knockdown inhibited the formation of cell cloning (Fig. 2F). These results indicated that knockdown CD24 inhibited cell proliferation in BC cells. Further, the wound-healing experiment was used to evaluate the migration ability of cells, and the experimental results showed that knockdown CD24 inhibited the wound healing ability of BC cells (Fig. 2G). In addition, transwell experiment was used to evaluate the invasion ability of cells, and the experimental results showed that the invasion ability of BC cells decreased after CD24 knockdown (Fig. 2H). This suggests that knocking down CD24 inhibits the migration and invasion of BC cells. In conclusion, we preliminatively concluded that knocking down CD24 in BC cells inhibits cell proliferation, migration, and invasion.

### CD24 promotes tumorigenesis of BC cells in vivo

**Fig. 3:**
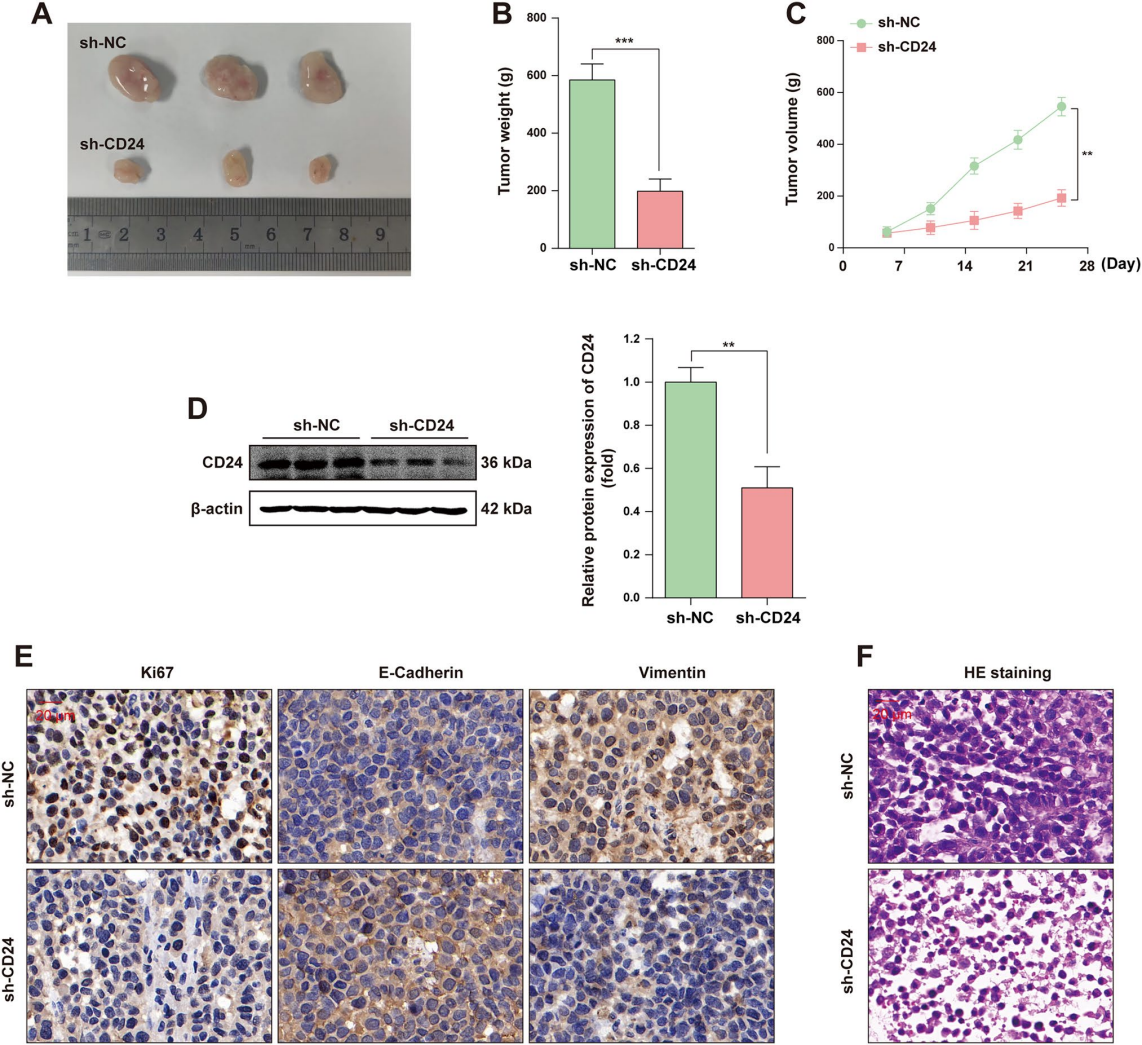
CD24 promotes tumorigenesis of BC cells in vivo. **A** MCF7 cells transfected with Ctrl shRNA and CD24 shRNA were injected subcutaneously into nude mice (n = 3) and euthanized by carbon dioxide 25 days after injection. **B** and **C** The mass and volume of subcutaneous heterograft tissue in nude mice were analyzed statistically. **D** WB assay was used to detect the expression level of CD24 in subcutaneous tumor tissue and the WB signals were normalized with β-actin. **E** The subcutaneous tumor sections of nude mice were stained by IHC with antibodies against Ki-67, E-Cadherin and Vimentin, and the representative results were shown. **F** Representative results of HE staining in subcutaneous tumor sections of nude mice. * *, *P* < 0.01; * * *, *P* < 0.001

To further determine the effect of CD24 on the malignant growth of BC in vivo, we conducted in vivo experiments. The MCF7 stable transfer cell line with CD24 knockdown and the control cell line were inoculated into the subcutaneous skin of nude mice respectively, and the subcutaneous tumors were harvested 25 days later for subsequent experimental analysis. The experimental results showed that CD24 knockdown effectively inhibited the occurrence of tumor in vivo, which was reflected in the significantly lighter weight and smaller volume of subcutaneous heterografts in nude mice in CD24 knockdown group compared with the control group (Fig. 3A, B, C). The expression level of CD24 in subcutaneous tumor tissues was further detected by WB experiment, and the results showed that the expression level of CD24 in subcutaneous tumor tissues of the control group was significantly higher than that of the experimental group (Fig. 3D). In addition, IHC experiments analyzed the expression levels of cell proliferation markers Ki-67 and metastasis markers E-Cadherin and Vimentin in subcutaneous tumor tissues, and the results showed that the expression levels of Ki-67 and Vimentin in the experimental group were down-regulated, while the expression levels of E-Cadherin were up-regulated (Fig. 3E). HE staining further indicated that the cell nuclei in the CD24 knockdown subcutaneous tumor tissue were relatively shallow and the cell arrangement was relatively regular (Fig. 3F). All in all, these results indicate that knockdown of CD24 in vivo inhibits the growth of subcutaneous heterograft tumor and reduces the malignant degree of BC.

### ZNF460 targets the promoter of CD24 to enhance its expression

**Fig. 4:**
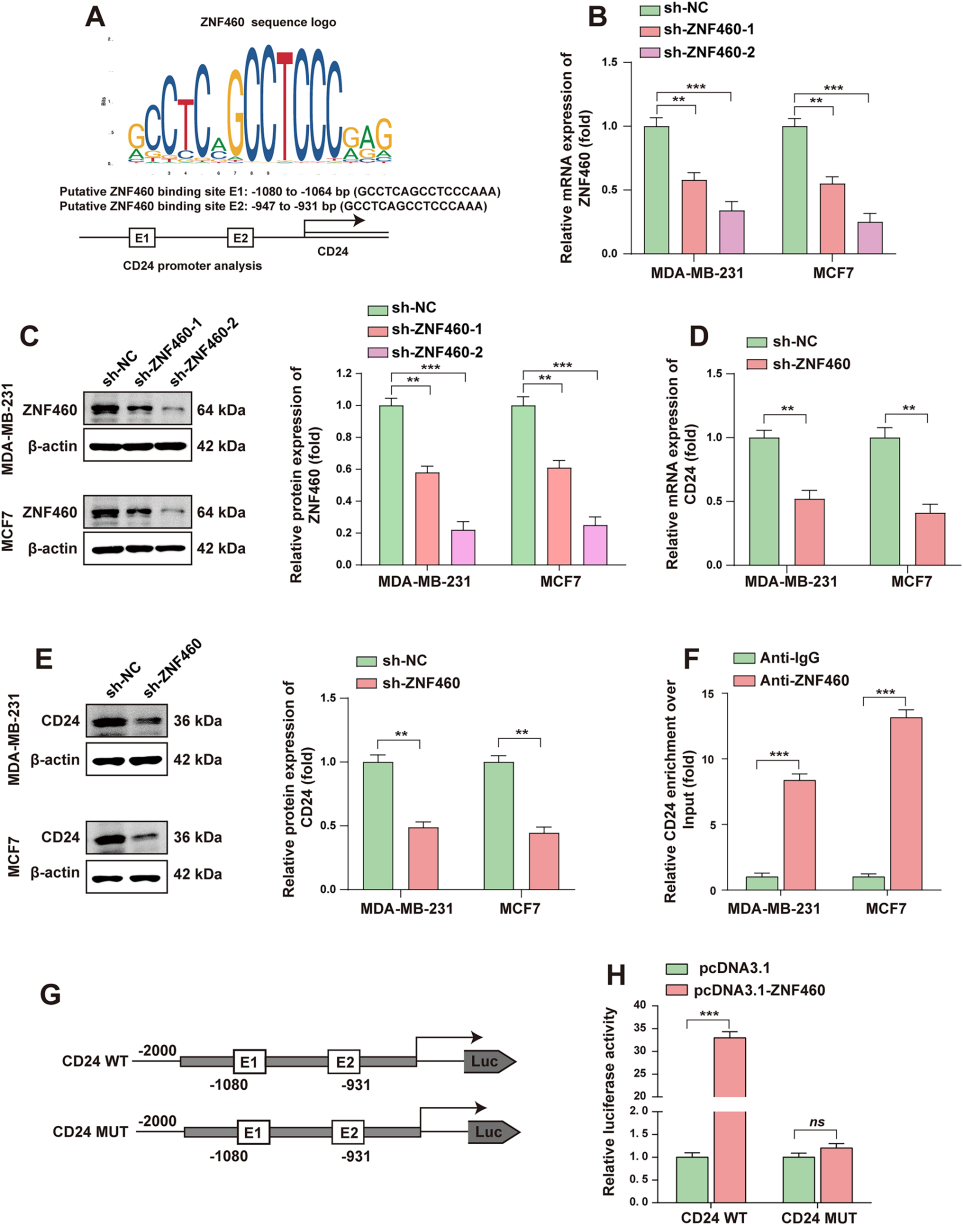
ZNF460 targets the promoter of CD24 to promote its expression. **A** JASPAR database (https://jaspar.genereg.net/) was used to predict the CD24 promoter transcription factors and shows ZNF460 binding sites. **B** RT-qPCR was used to detect the knockdown efficiency of ZNF460 mRNA in BC cells. **C** RT-qPCR assay was used to detect the effect of ZNF460 knockdown on CD24 mRNA levels. **D** WB assay was used to detect the effect of ZNF460 knockdown on CD24 mRNA levels. **E** The knockdown efficiency of ZNF460 protein in BC cells was measured by WB assay and the WB signals were normalized with β-actin. **F** The ChIP assay was used to verify the binding of ZNF460 as a transcription factor to the endogenous CD24 promoter region. **G** Construction diagram of wild type and mutant CD24 promoter luciferase reporter plasmid. **H** Dual luciferase reporter assay was used to verify the binding of ZNF460 to the CD24 promoter region. * *, *P* < 0.01; * * *, *P* < 0.001

In order to explore the CD24 molecular mechanism of regulation in the BC, UCSC website (https://genome.ucsc.edu/) is used to find the CD24 promoter regions, then JASPAR database (https://jaspar.genereg.net/) was used to predict the transcription factors. According to the predicted results, transcription factor ZNF460 with a high score was selected for follow-up study. We found two identical and ZNF460 binding sites located between -921 bp to -936 bp (GCCTCAGCCTCCCAAA) and -1054 bp to -1069 bp (GCCTCAGCCTCCCAAA) at the CD24 promoter, respectively (Fig. 4A). To investigate whether ZNF460 is involved in regulating CD24 expression, stable knockdown cell lines of ZNF460 were constructed (Fig. 4B, C). RT-qPCR and WB assay results showed that the mRNA and protein expression of CD24 in cells decreased after ZNF460 knockdown (Fig. 4D, E), suggesting that ZNF460 regulates the expression of CD24 in BC. Furthermore, chromatin immunoprecipitation (ChIP) was used to verify whether ZNF460 could bind the CD24 promoter, and the results showed that the concentration of CD24 in the ZNF460 antibody group was significantly higher than that in the control IgG (Fig. 4F). In addition, we constructed the wild-type and mutant luciferase reporter plasms in the CD24 promoter region (Fig. 4G), and the results of dual luciferase reporting experiments showed that ZNF460 could significantly activate the luciferase activity of wild-type CD24, but had no effect on the luciferase activity of mutant CD24 (Fig. 4H). In conclusion, we preliminarily concluded that ZNF460 acts as a transcription factor and promotes its transcription and protein expression by binding CD24 promoter.

### MiR-125a-5p regulates the expression of ZNF460 by targeting its 3’UTR

**Fig. 5:**
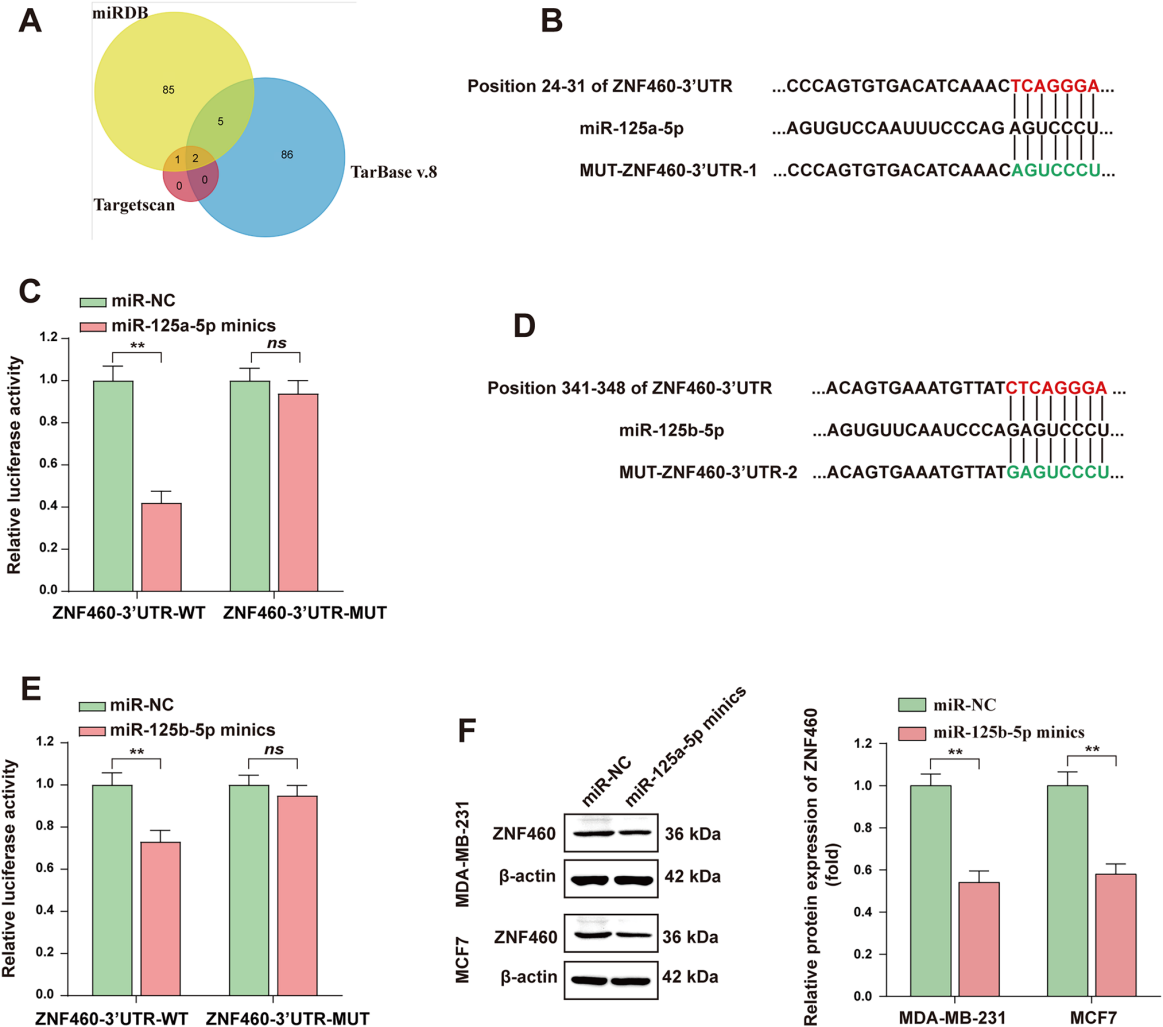
MiR-125a-5p regulates the expression of ZNF460 by targeting its 3’UTR. **A** The Venn diagram intersection shows the miRNAs predicted by Targetscan, miRDB, and TarBase v.8 that can target ZNF460. **B** Diagram of the constructed wild type (WT) and mutant (MUT) luciferase reporter plasmid of ZNF460 3’UTR containing miR-125a-5p binding sites. **C** Dual luciferase assay determined the binding of miR-125a-5p to the 3’UTR of ZNF460. **D** Diagram of constructed wild type and mutant luciferase reporter plasmids of ZNF460 3’UTR containing miR-125b-5p binding sites. **E** Dual luciferase assay determined the binding of miR-125b-5p to the 3'UTR of ZNF460. **F** WB assay was used to detect the expression level of CD24 after overexpression of miR-125a-5p in BC cells and the WB signals were normalized with β-actin. * *, *P* < 0.01; * * *, *P* < 0.001

Further, we explored the regulatory mechanism of ZNF460 in BC. MiRNA is involved in the expression of many genes in vivo and plays a biological function of promoting or suppressing cancer. We sought to find miRNAs that could regulate the expression of ZNF460. Three different websites were used to predict the miRNAs that can regulate ZNF460, including Targetscan, miRDB, and TarBase v.8, and it was found that the intersection of the predicted results of the three websites was miR-125a-5p and miR-125b-5p (Fig. 5A). To further identify miRNAs regulating ZNF460, We constructed the 3’UTR luciferase reporter plasmid of ZNF460 containing the miR-125a-5p binding site (Fig. 5B). The double luciferase reporter experiment results showed that miR-125a-5p mimics could significantly inhibit the luciferase activity of wild-type ZNF460 3’UTR. When the binding site of miR-125a-5p was mutated, the inhibition was removed on the luciferase activity of miR-125a-5p mimics on ZNF460 3'UTR (Fig. 5C). Similarly, we constructed the ZNF460 3'UTR luciferase reporter plasmid containing the miR-125b-5p binding site (Fig. 5D). The results of dual luciferase experiment showed that miR-125b-5p could partially inhibit the luciferase activity of ZNF460 3'UTR (Fig. 5E), but the inhibitory effect was not as obvious as that of miR-125a-5p. Therefore, we mainly explored the influence of miR-125a-5p on the expression of ZNF460. Further, miR-125a-5p mimics and its control were transfected into BC cells, and the expression level of CD24 protein was detected by WB assay. The results showed that transfection of miR-125a-5p mimics inhibited the expression of ZNF460 protein. The above experimental results indicated that miR-125a-5p regulates the expression of ZNF460 by targeting ZNF460.

### LINC00525 exerts its tumor-promoting effect in BC by targeting miR-125a-5p

**Fig. 6:**
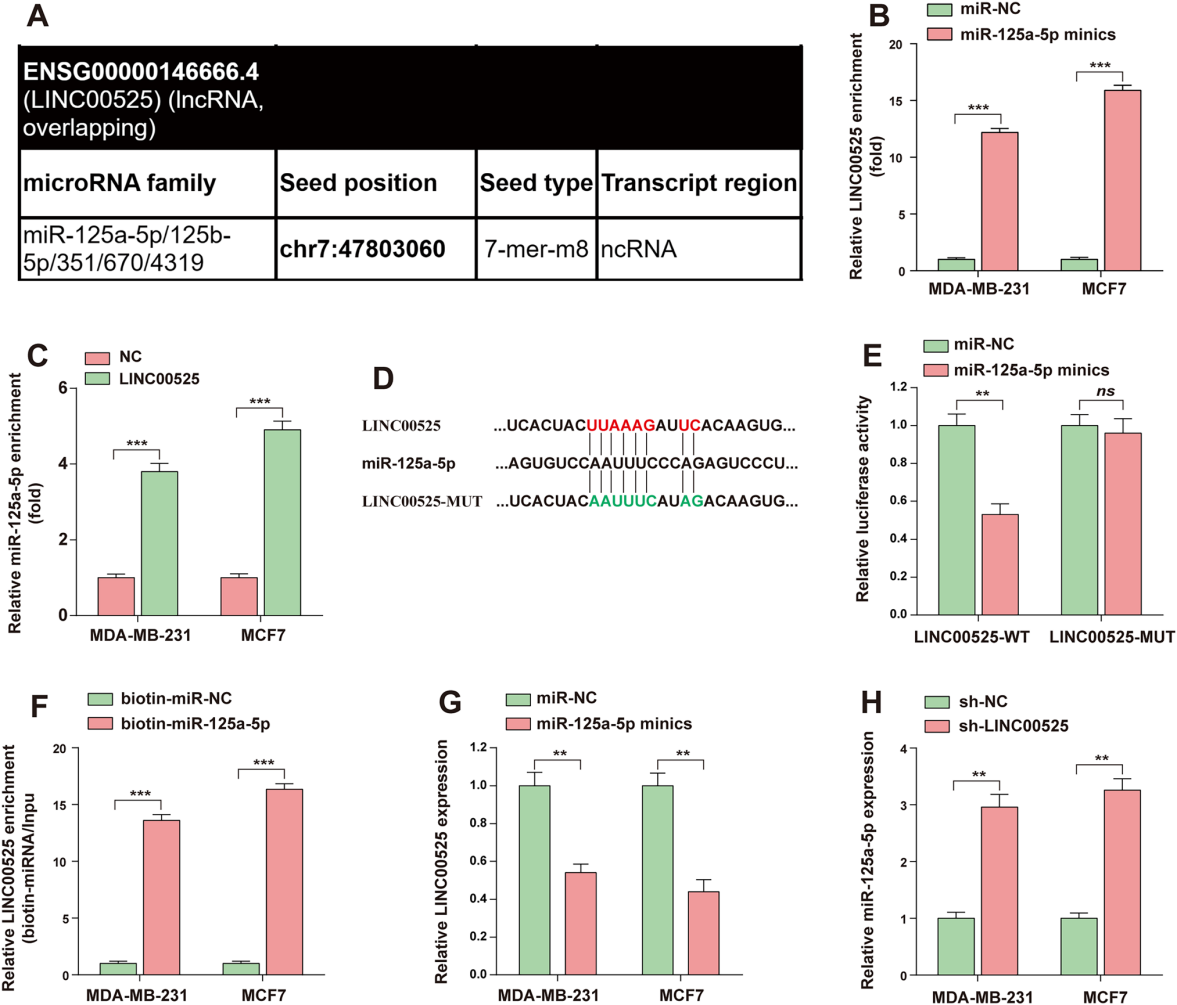
LINC00525 exerts its tumor-promoting effect in BC by targeting miR-125a-5p. **A** The miRcode database (http://www.mircode.org/) was used to predict LINC00525 as the complementary sequence of miR-125a-5p. **B** AGO2-RIP assay was used to determine LINC00958 enrichment in BC cells transfected with miR-125a-5p mimics or miR-NC. **C** The AGO2-RIP assay was used to determine miR-125a-5p enrichment in BC cells overexpressed with LINC00525 or control cells. **D** Construct a schematic of the LINC00525 luciferase reporter plasmid containing a putative binding sequence between miR-125a-5p and LINC00525. **E** Dual luciferase reporter assay was used to determine the binding of miR-125a-5p to LINC00525. **F** The spongy interaction between LINC00525 and miR-125a-5p was verified by biotin pull-down experiment. **G** BC cells were transfected with miR-125a-5p mimics or controls, and the expression level of LINC00525 was detected by RT-qPCR. **H** LINC00525 knockdown and RT-qPCR assay were used to detect the expression level of miR-125a-5p in BC cells. * *, *P* < 0.01; * * *, *P* < 0.001

It is well known that cytoplasmic lncRNAs can bind directly to miRNA and function as sponges or compete with ceRNAs to control the availability of miRNA for binding to their target mRNAs (Zhou et al. 2014). MiRcode (http://www.mircode.org/) database analysis found that LINC00525 can bind miR-125a-5p, which suggests the possibility of sponging action (Fig. 6A). The AGO2-RIP assay results showed that the enrichment of LINC00525 in the overexpression miR-125a-5p group was significantly higher than that in the control group (Fig. 6B); similarly, the enrichment of miR-125a-5p in the LINC00525 group was significantly higher than that in the control group (Fig. 6C). In addition, we constructed wild-type and mutant luciferase reporter plasmoids of LINC00525 containing miR-125a-5p binding sites (Fig. 6D), and the results of dual luciferase reporting experiments showed that miR-125a-5p could significantly inhibit luciferase activity of LINC00525 (Fig. 6E). Further, biotin-labeled miRNA pull-down assays revealed a significant increase in LINC00525 interactions in BC cells transfected with biotin-labeled miR-125a-5p compared to controls (Fig. 6F). Based on the above experimental results, we speculate that LINC0052 may act as a sponge for miR-125a-5p in BC. Then, our study found that transfection of miR-125a-5p simulators in BC cells down-regulated the expression of LINC00525 (Fig. 6G), and the expression level of miR-125a-5p increased after LINC00525 knockdown (Fig. 6H). The above experimental results indicate the spongy interaction between LINC00525 and miR-125a-5p.

## Discussion

More and more evidence confirms that the expression of CD24 in cancer is associated with poor prognosis and promotes malignant progression of tumors in a dose-dependent manner (Shapira et al. 2011). CD24 is one of P-selectin ligands, which is involved in the proliferation, invasion and metastasis of tumor cells through specific binding to P-selectin (Aigner et al. 1998; Carroll et al. 2018). Interestingly, targeting CD24 can enhance anti-tumor immune responses by inhibiting tumor-associated macrophages (TAM) (Zou et al. 2022). In addition, silencing CD24 can inhibit the growth of metastatic tumors (Overdevest et al. 2011). In this study, we found that CD24 was highly expressed in BC tissues and cell lines, and the high expression level of CD24 in BC was positively correlated with the invasion depth and TNM stage. In addition, high expression of CD24 in BC tissues was associated with poor prognosis, suggesting that it may be an independent prognostic indicator. Silencing CD24 inhibited the proliferation and migration of BC cells and inhibited the growth of BC subcutaneous transplanted tumors in nude mice. These results suggest that CD24 may play an important role in BC progression.

Under normal physiological conditions, ZNF460 is both a transcriptional activator and an oncogene, which is constitutionally activated in cancer and plays a crucial role in tumor progression (Hao et al. 2021). In addition to its traditional role in cancer cell proliferation, invasion, and migration, ZNF460 also promotes cancer development as a transcription factor by altering the expression of other genes in cancer cells (An and Liu 2023; Wang et al. 2021; Zou et al. 2021). In this study, we found that CD24 is a direct target of the transcription factor ZNF460. ZNF460 binds to prediction sites in the CD24 promoter region, and ZNF460 leads to significant induction of CD24 promoter activity, so the up-regulation of CD24 in BC is due in part to CD24 activation during tumor progression.

In this study, the interaction between miR-125a-5p and ZNF460 was first reported, and the mechanism of miR-125a-5p in BC cells was demonstrated. Many studies have reported the antitumor effects of miR-125a-5p in breast cancer, synovial sarcoma, and non-small cell lung cancer cells. In colorectal cancer cells, miR-125a-5p targets VEGFA, thereby inhibiting cell proliferation, migration, and invasion (Yang et al. 2018). The anticancer effect of miR-125a-5p can be achieved by targeting FUT4, which acts as a tumor driver for bladder cancer progression (Zhang et al. 2018). In addition, miR-125a-5p acts as a tumor suppressor for breast cancer by down-regulating BAP1 (Yan et al. 2018). The results of this study showed that miR-125a-5p was underexpressed in BC cells and targeted ZNF460 to regulate the proliferation, migration and invasion of BC cells, indicating that miR-125a-5p had significant anti-tumor effects in BC. These findings were consistent with previous studies on miR-125a-5p in BC.

Studies have shown that LINC00525 is highly expressed in a variety of cancers. The expression of LINC00525 is up-regulated in lung adenocarcinoma. On the one hand, LINC00525 inhibits the transcription of p21 gene by recruiting EZH2 to p21 promoter. On the other hand, LINC00525 reduces the stability of p21 mRNA through competitive binding with RBMS21 in the cytoplasm, and further promotes the proliferation of lung adenocarcinoma cells (Fang et al. 2021). Du et al. found that silencing LINC00525 inhibited the growth and epithelial-mesenchymal transformation of oral squamous cell carcinoma (Du et al. 2023). In addition, LINC00525 interacts with miR-31-5p and miR-125a-5p, acting as a carcinogenic molecule in chordoma (Gong et al. 2021). Our study found that LINC00525 is up-regulated in BC, LINC00525 acts as ceRNA to increase the expression of ZNF460 mRNA by competing with miR-125a-5p to maintain CD24 protein to promote the proliferation and transfer of BC.

## Conclusion

This study identified CD24 as an oncogenic protein in BC. ZNF460, as a transcription factor, up-regulates the expression of CD24 through transcriptional regulation. MiR-125a-5p can regulate the expression of ZNF460 by targeting the 3’UTR of ZNF460. In addition, LINC00525 can promote the proliferation and metastasis of BC through sponge absorption of miR-125a-5p. Our results support the notion that CD24 plays a key role in the progression of BC and demonstrate that CD24 is a potential effective target for the treatment of BC.

### Supplementary Information

Below is the link to the electronic supplementary material.

Supplementary file1 (DOCX 17 KB)

## Data Availability

The data used to support the findings of this study are available from the corresponding author upon reasonable request.
