# Supplementary material for: LINC00525 enhances ZNF460-regulated CD24 expression through the sponge miR-125a-5p to promote malignant progression of breast cancer
Source: J Cancer Res Clin Oncol. 2024 Jun 24;150(6):317. doi: 10.1007/s00432-024-05830-2 (PMC11196364; doi:10.1007/s00432-024-05830-2)
Supplement: Supplementary file 1 — Supplementary file1 (DOCX 17 KB) [file 432_2024_5830_MOESM1_ESM.docx]

**Supplementary Tables**

**Table 1.** Primer information required for RT-qPCR process.

| **Primer name** | **Primer sequence (5′–3′)** |
| --- | --- |
| CD24-Forward | TTCTCCAAGCACCCAGCA |
| CD24-Reverse | TGGAATAAATCTGCGTGGGTA |
| ZNF460-Forward | CTCATTCGACACTTCAACATCC |
| ZNF460-Reverse | GTGGATGCTAAAGTGTCGAATC |
| β-Actin-Forward | CATGTACGTTGCTATCCAGGC |
| β-Actin -Reverse | CTCCTTAATGTCACGCACGAT |
| LINC00525-Forward | GTGGATGTACGGTGCAAGGA |
| LINC00525-Reverse | TGCAACTACGACCCCGAAAA |
| miR-125a-5p-Forward | TCCCTGAGACCCTTTAAC |
| miR-125a-5p-Reverse | TTTGGCACTAGCACATT |
| U6-Forward | CTCGCTTCGGCAGCACA |
| U6 -Reverse | AACGCTTCACGAATTTGCGT |

Table 2. Relationship between CD24 expression and clinicopathological features in 16 HCC tissues

| Clinicopathologic parameters | n | CD24 expression | | p |
| --- | --- | --- | --- | --- |
|  |  | low | high |  |
| All cases | 16 | 5 | 11 |  |
| Age |  |  |  | 0.698 |
| ≤ 55 | 10 | 4 | 6 |  |
| >55 | 6 | 1 | 5 |  |
| Histological type |  |  |  | 0.045 |
| Luminal | 11 | 4 | 7 |  |
| HER2 postive | 3 | 1 | 2 |  |
| Triple negative | 2 | 0 | 2 |  |
| T classification |  |  |  | 0.014 |
| T1-T2 | 12 | 3 | 9 |  |
| T3-T4 | 4 | 2 | 2 |  |
| Lymph node metastasis |  |  |  | 0.195 |
| no | 9 | 4 | 5 |  |
| yes | 7 | 1 | 6 |  |
| TNM stage |  |  |  | 0.032 |
| I+II | 10 | 2 | 8 |  |
| III+IV | 6 | 3 | 3 |  |

**Table 3.** Base sequence of Chip

| **shRNA name** | **shRNA sequence** |
| --- | --- |
| CD24-Forward | TAGGCTGGAGTGCAGTGGCAT |
| CD24 -Reverse | TGCTTAGAGCTCGTTCAAGAAT |

**Table 4.** Base sequence of miRNA

| **miRNA name** | **miRNA regulate sequence (5′–3′)** |
| --- | --- |
| miR-125a-5p-NC | UUCUCCGAACGUGUCACGUTT- |
| miR-125a-5p-mimic | UCUCCCCUUGUAACCAGACCUG |
| miR-125b-5p-NC | UUCUCCGAACGUGUCACGUTT |
| miR-125b-5p-mimic | UCCCUGAGACCCUAACUUGUGA |

**Table 5.** Base sequence of shRNA

| **shRNA name** | **shRNA sequence** |
| --- | --- |
| sh-CD24-1 | TGCCTCGACACACATAAAC |
| sh-CD24-2 | TTGCATTGACCACGACTAA |
| sh-ZNF460-1 | GCGACAGCTGATGGTATTTGT |
| sh-ZNF460-2 | GCATAACAAGAGCCACAATGA |
| sh-LINC00525-1 | AUUAAGGUUCUCAAUACAGUA |
| sh-LINC00525-2 | UAUAUAUUUUGUUGUAUUCAU |
